# Deep learning to predict extrapancreatic perineural invasion at CT images

**DOI:** 10.1080/07853890.2025.2568116

**Published:** 2025-12-12

**Authors:** Zhenghua Cai, Liwen Zou, Qi Li, Jun Chen, Yudong Qiu, Jingjing Ji, Liang Mao

**Affiliations:** ^a^ Department of Pancreatic and Bariatric Surgery, Nanjing Drum Tower Hospital, The Affiliated Hospital of Nanjing University Medical School, Nanjing City, Jiangsu Province, China; ^b^ Department of Mathematics, Nanjing University, China; ^c^ Department of Pathology, Nanjing Drum Tower Hospital, The Affiliated Hospital of Nanjing University Medical School, Nanjing City, Jiangsu Province, China; ^d^ Department of Anesthesiology, Nanjing Drum Tower Hospital, The Affiliated Hospital of Nanjing University Medical School, Nanjing City, Jiangsu Province, China

**Keywords:** Pancreatic ductal adenocarcinoma, extrapancreatic nerve plexus, extrapancreatic perineural invasion, segmentation, deep learning model

## Abstract

**Background:**

Extrapancreatic perineural invasion (EPNI) was an adverse prognostic factor in patients with pancreatic ductal adenocarcinoma (PDAC) and responsible for positive resection margin. This study aimed to develop an automatic model for segmenting the extrapancreatic nerve plexus and diagnosing EPNI.

**Methods:**

In this retrospective study, patients diagnosed with PDAC who underwent enhanced computer tomography scans between August 2018 and December 2022 were enrolled. These cases were divided into training sets with radiological EPNI labels, and validation sets with pathological EPNI labels. The extrapancreatic nerve plexus was segmented first *via* the nnUNet network and attention mechanism under the background of segmentation of PDAC and adjacent vessels. A 2D classifier was applied to diagnose EPNI based on the segmentation of the nerve plexus. The Dice similarity coefficients (DSCs), receiver operating characteristic (ROC) curve, and diagnostic accuracy were employed to evaluate the performance of the model.

**Results:**

A total of 332 consecutive patients were enrolled and classified into the training (*n* = 282) and validation (*n* = 50) sets. Patients diagnosed with EPNI accounted for 177 of the 332 patients (53.3%). On the one hand, the model showed modest DSCs in segmenting nerve plexus around celiac axis (CA), superior mesenteric artery (SMA), and common hepatic artery (CHA), which were 60, 68.2 and 35.7%, respectively. On the other hand, the model had a favorable performance in diagnosing EPNI; the accuracy and areas under the ROC curve were 0.797, 0.8 in training set and 0.72, 0.85 in the validation set.

**Conclusions:**

The fully automatic deep learning model for segmenting the nerve plexus and diagnosing EPNI was a novel and promising tool. Further studies are required to improve the model performance.

## Introduction

Pancreatic ductal adenocarcinoma (PDAC), the most virulent neoplasm within the gastrointestinal tract, exhibits aggressive biological behavior and is projected to become the second leading cause of cancer-related mortality by 2030 [1,2,]. To improve the prognosis of PDAC, extensive research has been undertaken, including early detection, patient stratification based on biological behaviors, advancements in surgical methodologies, and refinement of chemotherapeutic protocols [3–6]. Of these endeavors, the preoperative assessment of biological characteristics is paramount, including the concentration of carbohydrate antigen 19-9 (CA19-9), the presence of regional lymph node metastasis, and the focus of our investigation, extrapancreatic perineural invasion (EPNI) [7,8].

Anatomically, the pancreas is situated adjacent to three principal arteries: the celiac axis (CA), superior mesenteric artery (SMA), and common hepatic artery (CHA), which are accompanied by nerve plexuses originating from the abdominal sympathetic and parasympathetic trunks. Given the neural tropism of PDAC cells, the incidence of EPNI is approximately 69%, as reported by Zuo et al. [9]. The mechanisms of EPNI include direct invasion, infiltration of surrounding vascular structures, and intratumoral perineural invasion [10]. The consensus holds that EPNI is closely correlated with positive surgical resection margins, reduced recurrence-free intervals, and diminished overall survival rates [11]. Consequently, the identification of EPNI during diagnosis and the selection of appropriate therapeutic regimens are important for improving long-term outcomes.

Presently, preoperative computed tomography (CT) evaluation primarily focuses on tumor resectability, lymph node status, and distant metastasis, with relatively few radiologists and surgeons focusing on EPNI. Furthermore, the varied radiological manifestations of EPNI contribute to significant discrepancies in diagnostic accuracy between pancreatic centers and radiologists with different levels of expertise. Recently, deep learning networks have garnered the interest of medical professionals in the domains of early diagnosis, tumor segmentation, and prognostic forecasting [12]. These deep learning models are characterized by their rapidity and consistency, and have demonstrated superior performance and efficiency compared to traditional methods. Hence, our study aimed to develop and validate a fully automatic segmentation model for the extrapancreatic nerve plexus and to predict EPNI on CT images.

## Methods

### Patients and ethics

This single-center retrospective study was reviewed and approved by the Ethics Committee of Drum-Tower Hospital Affiliated to Nanjing University Medical School (No. 2023-161). All the patients signed an informed consent form. Additionally, This study complied with the requirements of the Helsinki Declaration.

**Figure 1.**
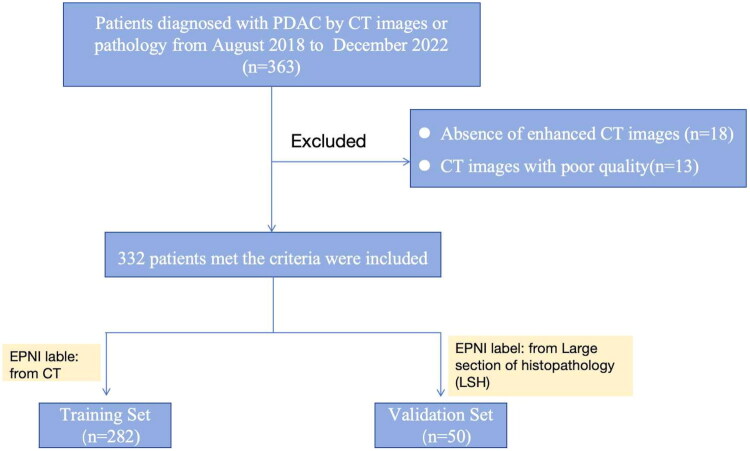
Flowchart of enrollment.

The inclusion criteria was patients who diagnosed with PDAC from August 2018 to December 2022, supported by radiological or pathological evidence. Exclusion criteria included the lack of enhanced CT images or CT images of poor quality that could not be used for further segmentation. A STROBE diagram is presented in Figure 1. Ultimately, 363 patients were enrolled, but 31 were excluded due to the absence of CT images or images that were too indistinct to segment the relevant structures.

### CT scanning protocol and data collection

CT scans were performed using a multi-slice spiral CT scanner (Light Speed, VCT, or Discovery HD750, GE Healthcare, United States). The parameters for the enhanced CT scans were set as follows: tube voltage at 120 kVp, tube current at 250–300 mAs, slice thickness and spacing both at 1.25 mm, matrix size at 512 × 512, rotation time at 0.6 s, pitch at 1.375, and field of view ranging from 35 to 40 cm. All patients were required to abstain from food for a minimum of 6–8 h prior to the CT scan. During the procedure, patients were positioned supine with their arms elevated, and the scan covered the area from the dome of the diaphragm to the bilateral renal pelvis. Following the CT plain scan, an iodinated contrast agent was administered intravenously at a dose of 1.5 ml/kg (Omnipaque 350 mg/mL, GE Healthcare, United States) and infused at a rate of 3.5 ml/s *via* the Medrad Stellant CT syringe system (located at One Medrad Drive, Indianola, Pennsylvania, United States). After contrast agent injection, scans were conducted during the arterial, pancreatic parenchymal, portal vein, and delayed phases.

CT images of the patients were retrieved from the picture archiving and communication system (PACS) in Digital Imaging and Communications in Medicine (DICOM) format, encompassing the plain, arterial, pancreatic parenchymal, portal, and delayed phases. These images were then converted into NIfTI format for subsequent analysis.

Meanwhile, the clinical data of the patients were retrieved from the hospital information system, including age, sex, body mass index, serum carbohydrate antigen 199 (CA19-9) concentration, tumor size, and related radiological indices.

To develop a fully automated model to segment the nerve plexus and diagnose EPNI, patients were classified into two groups. The training set included patients diagnosed with EPNI based on radiological signs rather than pathological results, while the validation set included patients whose specimens were subjected to large section histopathology (LSH) for the diagnosis of EPNI. Compared with routine sectioning techniques, LSH requires a complete tissue block to better display the relationship between the tumor and surrounding tissues, and to observe the resection margin. The detailed procedures of LSH are depicted in the Supplementary method and Figure (Figure S1).

Of the five collected scanning phases, the arterial phase was used for subsequent segmentation and analysis. The annotation included the nerve plexus around the CHA, SMA and CA. The annotated areas of nerve plexuses originated from the targeted artery to the edge of the pancreas, marking all visible adipose tissues based on the distribution patterns of the nerve plexus. For the nerve plexus around the CHA, the annotation ranged from the origin of the CHA to the junction with the proper hepatic artery. For the nerve plexus around the CA, it originated from the root of the CA to its downstream branches, CHA, and splenic artery. For the nerve plexus around the SMA, it originated from the root of the SMA to the second branch of the jejunal artery. Meanwhile, the ganglion located within the triangle area surrounded by the CA, SMA, and portal vein was also marked. In cases of EPNI, the invaded areas were manually annotated. EPNI was considered when streaky and strand-like intensity structures or irregular soft tissues extending from the lesion were observed in the adipose tissues around the SMA, CHA, and CA [13,14].

The aforementioned work was conducted by two specialists, Liang Mao, with 18 years of experience, and Zhenghua Cai, with 8 years of experience. In disagreement of EPNI diagnosis, Dr. Yudong Qiu, who had 30 years of experience, determined the results. We calculated the inter-observer variability based on the dice similarity coefficients (DSCs) and detection rate (DR) metrics. The DSC metric measures the pixel-wise overlap of the annotations on the nerve plexus between the two specialists. The DR metric evaluated the consistency of the positioning between the two annotators. Examples of the annotations were shown in Figure S2.

### Development and validation of deep learning model

**Figure 2.**
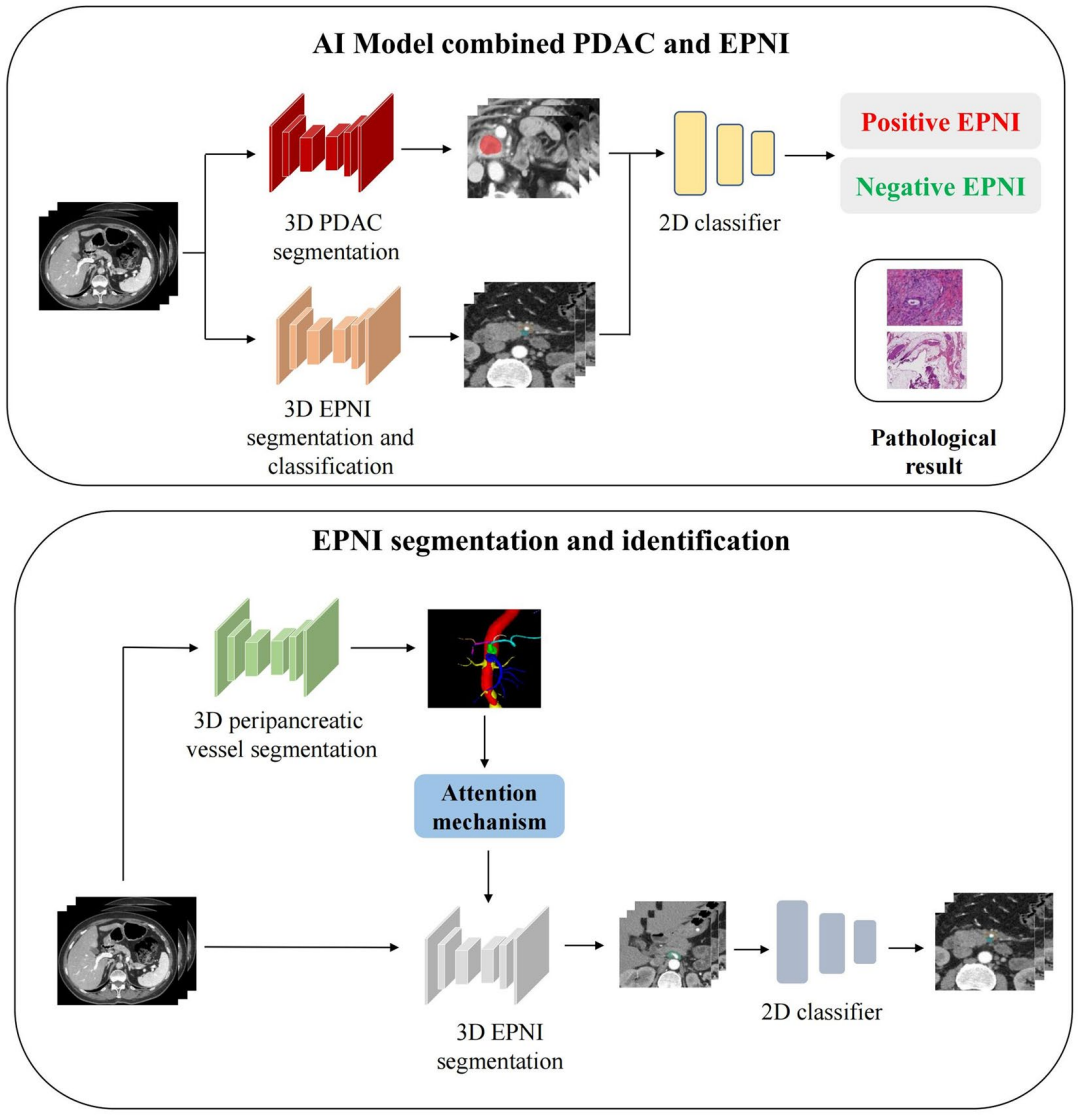
Flowchart of segmentation and classification by deep learning model.

In order to segment the extrapancreatic nerve plexus, we initially segmented the tumor and nearby vessels. The PDAC segmentation utilized the second stage of a pre-trained model built on a standard 3D U-Net framework, incorporating an encoder for feature extraction, a decoder for generating segmentation probability maps, and skip connections between encoder and decoder layers at varying resolutions [15,16]. The encoder comprised nine convolutional blocks, each containing two successive 3 × 3 × 3 convolution layers, instance normalization, and leaky ReLU activation, with dropout layers integrated for regularization. The decoder consisted of eight convolutional blocks that processed features from the preceding block, upsampled them *via* a 2 × 2 × 2 transposed convolution, and merged them with skip connections from the encoder. For peripancreatic vessel segmentation, we leveraged a pretrained nnUNet-based model [17,18]. The EPNI segmentation followed the same backbone as the vessel network but incorporated an additional attention mechanism to enhance detection accuracy by embedding vascular information [19]. The EPNI classification model adapted a ResNet101 architecture, featuring 101 convolutional layers and 33 residual blocks, culminating in global average pooling and a fully connected layer for score prediction [20]. We enhanced the final residual block with a texture-encoding layer, fusing its output with deep features *via* concatenation before classification. Figure 2 illustrated the segmentation workflow.

During the proprocessing process, CT images were normalized to a [0, 1] range by first extracting all foreground voxels from the training set. Intensity values were clipped at the 0.5th and 99.5th percentiles to reduce outlier influence, followed by normalization using the global foreground mean and standard deviation. Spatial resampling was performed by computing the median voxel spacing across the training data and resampling all cases to this target resolution using third-order spline interpolation. The PDAC, peripancreatic vessel, and EPNI segmentation networks were each trained for 1,000 epochs, while the classification network underwent 500 epochs. Optimization was performed using stochastic gradient descent with Nesterov momentum (*μ* = 0.99) and an initial learning rate of 0.01, which was decayed following a polynomial schedule. The training set (*n* = 226 patients, 80%) was used for model training, with the remaining 20% (*n* = 56) reserved for validation and hyperparameter tuning to select optimal model checkpoints.

### Statistics analysis

Statistical analyses were conducted using IBM SPSS Statistics for Windows version 27.0 software (SPSS Inc.). Variables with a normal distribution are presented as mean ± standard deviation (SD) and compared using the independent *t* test. When the variables exhibited a skewed distribution, the Mann-Whitney *U* test was employed, and the results were expressed as medians (interquartile range, IQR). Categorical variables were compared using the *χ*^2^ test. The performance of the model was assessed using a receiver operating characteristic (ROC) curve.

## Results

### Baseline characteristics

Among the 363 patients diagnosed with PDAC *via* CT or pathology, 332 consecutive patients admitted between August 2018 and December 2022 were enrolled in accordance with the established inclusion and exclusion criteria. The training set comprised 282 cases with an EPNI label diagnosed by CT, whereas the remaining 50 cases with an EPNI label confirmed by LSH were categorized into the validation set. As indicated in Table 1, there were no significant differences in average age (*p* = 0.17), gender (*p* = 0.509), median BMI values (*p* = 0.574), median CA19-9 concentration (*p* = 0.771), median tumor size (*p* = 0.276), location of the lesions (*p* = 0.545), peri-pancreatic arteries (*p* = 0.6), and vein invasion (*p* = 1.0). However, the proportion of EPNI differed significantly between the two groups (57.8 vs. 28%, *p* = 0.001).

**Table 1.** Baseline characteristics of enrolled patients.

Characteristics	Training set (*n* = 282)	Validation set (*n* = 50)	*p* value
Age (year)	65.5 ± 10.6	64.4 ± 10.9	0.17
Gender (M/F)	155/127	30/20	0.509
BMI (kg/m^2^)	23.5 (21.5, 25.4)	23.2 (21.4, 25.1)	0.574
CA19-9 concentration (ng/ml)	131.2 (19.2, 283.2)	104.5 (23.3, 302.1)	0.771
Tumor size (cm)	2.6 (2, 3.2)	2.7 (2.1, 3.3)	0.276
Location of the lesion			0.545
Head	226	42	
Neck	50	8	
Body and Tail	6	0	
Proportion of EPNI, *n* (%)	163 (57.8%)	14 (28%)	0.001
Peri-pancreatic artery invasion, *n* (%)	7 (2.4%)	0	0.6
Peripancreatic vein invasion, *n* (%)	8 (2.8%)	1 (2%)	1.0

* BMI, body mass index; CA19-9, carbohydrate antigen 19-9; EPNI, extrapancreatic perineural invasion.

### The inter-observer variability and DSCs of deep learning model in segmenting nerve plexuses

After matching the annotations of the two specialists, it can be observed that the DSCs of the annotations was 0.638 for nerve plexus around CHA, 0.838 for CA, and 0.976 for SMA. The DR metric was 0.918 for nerve plexus around CHA, 0.972 for CA, and 1.0 for SMA, which demonstrated satisfactory inter-observer variability.

**Figure 3.**
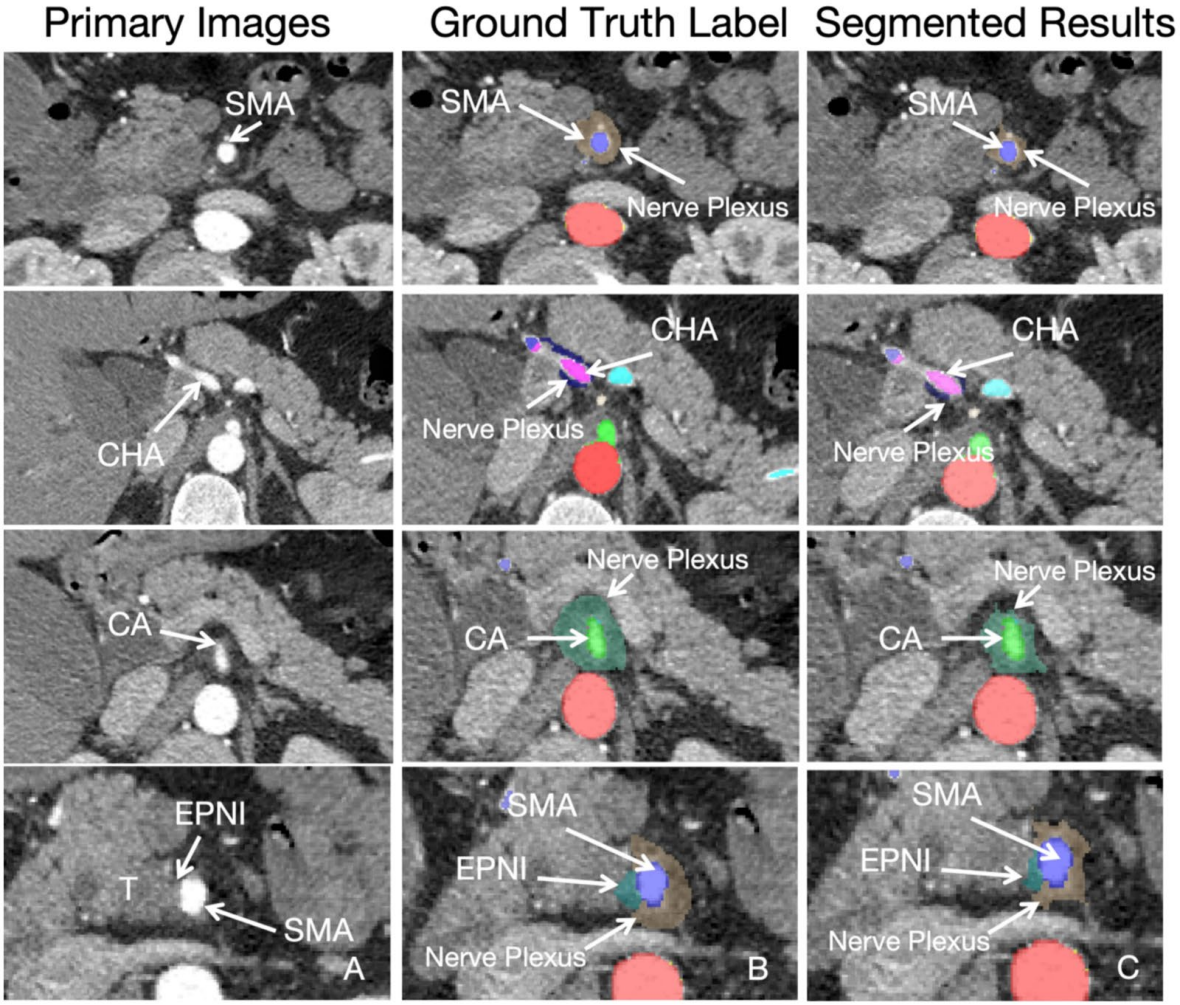
The segmented results of deep learning models. A, B, C represented the primary images, ground-truth labels and segmented results by deep learning model.

As shown in Table 2, the mean DSCs, 50% annotated area detection rate, and 10% annotated area detection rate for nerve plexus segmentation around the SMA were 68.2, 92.6, and 98.9%, respectively. Similarly, the values were 60, 80.9, and 95.6% for nerve plexus segmentation around the CA; 35.7, 55.1, and 87.1% for nerve plexus segmentation around the CHA; and 34.2, 45.9, and 76.4% for EPNI areas. The segmented results of the deep learning models were shown in Figure 3.

**Table 2.** Performance of the deep learning model in segmenting extrapancreatic nerve plexuses.

Case	DSCs	50% area detection rate	10% area detection rate
Nerve plexus around SMA	68.2%	92.6%	98.9%
Nerve plexus around CA	60%	80.9%	95.6%
Nerve plexus around CHA	35.7%	55.1%	87.1%
EPNI area	34.2%	45.9%	76.4%

* DSCs, dice similarity coefficients; CA, celiac axis; SMA, superior mesenteric artery; CHA, common hepatic artery.

EPNI, extrapancreatic perineural invasion.

### Diagnostic accuracy of EPNI based on CT images

Since the label provided in the training set was based on radiological features, its accuracy was further confirmed in the validation set using LSH. Table 3 indicated that out of the 50 patients in the validation set, 16 were diagnosed with EPNI based on CT images. Of these 16 patients, 14 were confirmed by pathological diagnosis, resulting in an overall radiological diagnostic accuracy of 87.5%. In subgroup analysis, the accuracy of CT diagnosis was 66.6% when EPNI presented as a streaky and strand-like intensity structure, and 92.3% when it presented as irregular soft tissue.

**Table 3.** Accuracy of radiological diagnosis of EPNI in validation set.

Radiological characteristics of EPNI	EPNI cases (pathology)	EPNI cases (CT)	Accuracy
Streaky and strand-like intensity structure (case)	2	3	66.6%
Irregular soft tissues (case)	12	13	92.3%
Total (case)	14	16	87.5%

* EPNI, extrapancreatic perineural invasion.

### Performance of the deep learning model in diagnosing EPNI

**Figure 4.**
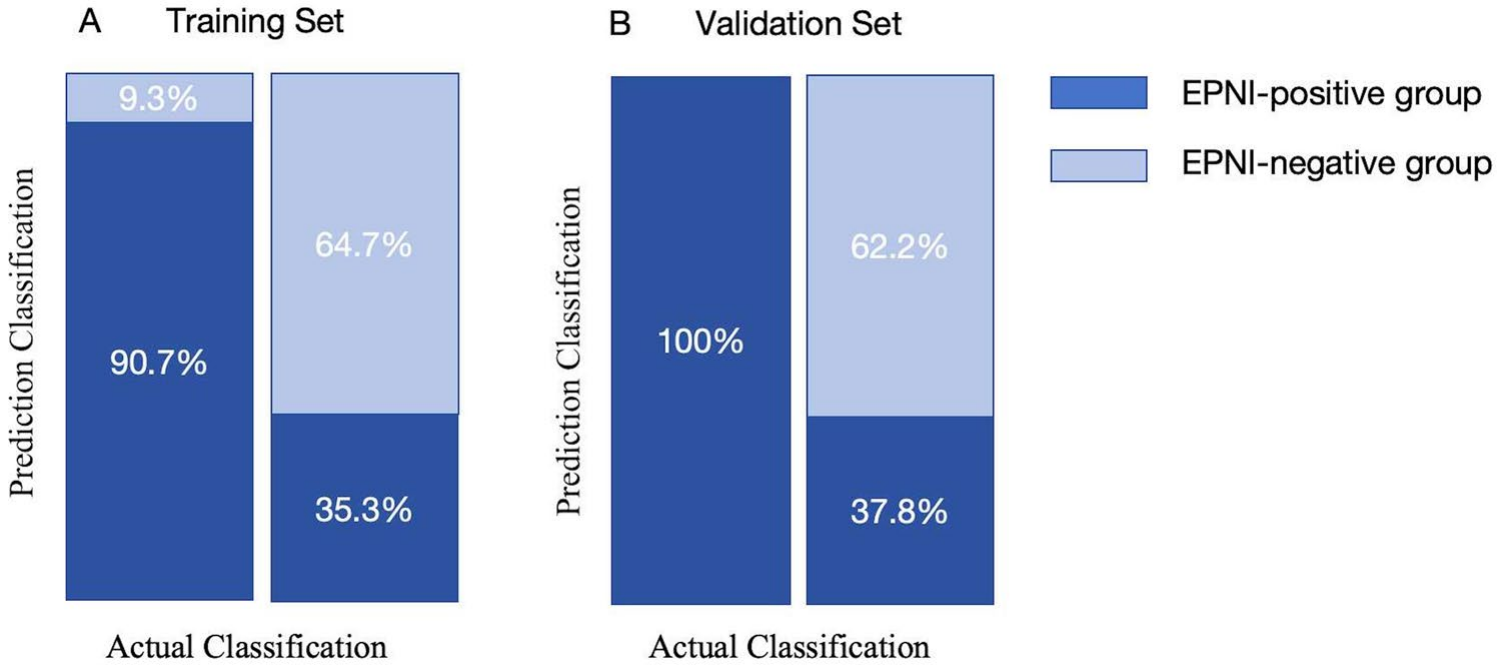
Performance of the deep learning model in diagnosing EPNI.

The deep learning model achieved high accuracy in distinguishing patients with and without EPNI (Table 4). The AUC was 0.8 (95%CI 0.728–0.826) in the training set and 0.85 (95%CI 0.75–0.898) in the validation set (Figure S3). Additionally, the sensitivity, specificity, and accuracy of the model were 90.7% (95%CI 85.4–94.3%), 64.7% (95%CI 55.8–72.7%), and 0.797, respectively, in the training set and 100% (95%CI 77.2–100%), 62.2% (95%CI 48.8–78.2%), and 0.72 in the validation set. Furthermore, as illustrated in Table 5, the accuracy of identifying EPNI around CA, SMA, and CHA was 0.851, 0.809, and 0.936 in the training set, and 1.0, 0.765, and 0.96 in the validation set, respectively. As indicated in Figure 4, the model was capable of accurately classifying 90.7 and 64.7% of the patients in the training set into EPNI-positive and-negative groups, respectively. Additionally, in the validation set, 100 and 62.2% of patients were correctly classified.

**Table 4.** Performance of deep learning model in EPNI diagnosis.

	TP (n)	FP (n)	FN (n)	TN (n)	Sensitivity	Specificity	Accuracy	AUC
Training set (*n* = 282)	148	42	15	77	90.7% (85.4%-94.3%)	64.7% (55.8%-72.7%)	0.797	0.80 (0.728–0.826)
Validation set (*n* = 50)	13	14	0	23	100% (77.2%-100%)	62.2 (48.8%-78.2%)	0.72	0.85 (0.75–0.898)

* TP, ture positive; FP, false positive; FN, false negative; TN, ture negative; AUC, areas under curve.

**Table 5.** Diagnostic accuracy of deep learning model in terms of different target areas.

	Nerve plexus around CA	Nerve plexus around SMA	Nerve plexus around CHA
Training set	85.1%	80.9%	93.6%
Validation set	100%	76.5%	96%

* CA, celiac axis; SMA, superior mesenteric artery; CHA, common hepatic artery.

## Discussion

To the best of our knowledge, this was the first study to segment the extrapancreatic nerve plexus and diagnose EPNI using a fully automated deep learning model. Encouragingly, the deep learning model demonstrated satisfactory Dice Similarity Coefficients (DSCs) in segmenting the nerve plexus around the CHA, SMA and CA. Moreover, the model exhibited a favorable performance in discriminating between EPNI-positive and EPNI-negative cases in both the training and validation sets.

Many studies have investigated the risk factors for the survival of patients with PDAC, including regional lymph node metastasis, invasion of the peripancreatic vessels, positive resection margins, perineural invasion, and EPNI [21–23]. Considerable efforts have been made to predict these prognostic factors more quickly and precisely preoperatively, thus several artificial intelligence models have been established [24,25]. However, a comprehensive review of recent reports revealed a lack of EPNI prediction models, which adds novelty and challenges to our study.

From an anatomical standpoint, seven extrapancreatic nerve plexuses create a complex network that regulates the function and sensation of the pancreas [26]. We omitted the splenic artery nerve plexus (PLspa) and the hepatoduodenal ligament nerve plexus (PLhdl), focusing instead on the remaining five pathways as the target area for constructing a deep learning model. The rationale is that the PLspa is dissected concurrently during left pancreatectomy, and if the PLhdl was invaded, the lesion is deemed unresectable [7]. We selected the other five pathways for segmentation because their excessive clearance could lead to severe metabolic disorders. Hang et al. reported that the routine “Triangle Operation” added unnecessary complexity to the procedure and increased the incidence of postoperative complications, yet no better prognosis was observed [27]. Kuroki et al. found that semi-circumferential or more extensive nerve dissection around the CA or SMA did not contribute to extending overall survival, with 93.6% of these patients suffering from refractory diarrhea [28]. Inoue et al. also concluded that extensive dissection around the SMA should be performed with great selectivity, although postoperative diarrhea can be managed with opioid assistance [29]. Given the absence of an international consensus on the dissection of the extrapancreatic nerve plexus, various studies have indicated that precise rather than extensive clearance of the nerve plexus could improve both short-term and long-term outcomes for patients with pancreatic ductal adenocarcinoma (PDAC) [30,31]. The essence of precise dissection hinges on accurately diagnosing extrapancreatic nerve involvement (EPNI) through CT or MR imaging.

Similar to assessing the status of lymph nodes, it is widely recognized that CT is the first choice for diagnosing EPNI [13,14]. However, diagnostic accuracy varies among radiologists due to the atypical radiological appearance of EPNI. As reported, by extracting invisible computer-associated features, deep learning or radiomics can achieve consistent accuracy, surpassing that of doctors in certain areas. However, Bian et al. believed that it was difficult for radiomics to replicate the previous results and demanded a large number of manual annotations [32]. Here, we adopted a deep-learning network for segmentation and diagnosis.

Before manually annotating the extrapancreatic nerve plexus, our team had already developed an automatic segmentation model for the pancreas and peripancreatic vasculature, as well as a labeling model [15,17,33]. As previously mentioned, the nerve plexus, along with lymph nodes and vessels, is enveloped in the fat tissues situated between the pancreas and the main abdominal arteries. Utilizing these two models and ample manual annotations, we can directly segment the nerve plexus using nnUNet, enhanced with an attention mechanism. As anticipated, our model achieved acceptable DSCs for the segmentation of the extrapancreatic nerve plexus area, with values of 68.2, 60, and 35.7% for the regions around the SMA, CA, and CHA, respectively. The detection rates for the 50% target area were 92.6, 80.9, and 55.1% for the SMA, CA, and CHA, respectively. It is evident that the segmentation DSCs for the nerve plexus around the CHA were the lowest among the three areas, which may be attributed to anatomical complexity or variability, labeling ambiguity in the training data, and algorithmic limitations. Moving forward, we plan to optimize the model by advancing its architecture and refining post-processing techniques.

The provided dataset has served as an essential foundation for extracting deep learning features for subsequent classification. In our cohort, the proportion of EPNI was 53.3%, in contrast to the 69% reported in Zuo et al.’s study [9]. The difference between the two studies lies in the fact that our research primarily focused on pancreatic cancer located in the head and neck, whereas Zuo et al. reported the overall incidence, including tumors in the head, neck, body, and tail. The nerve plexus surrounding the splenic artery was often difficult to disentangle from the tumor invasion. Notably, due to the destruction of the resection margin caused by routine fixation procedures during the preparation of specimen slices, pathologists were seldom able to make an accurate diagnosis of EPNI, particularly when the tumor presented with a small invasive lesion [34]. Consequently, the ground-truth label in the training set was based on radiological diagnosis. However, there was an inevitable discrepancy between radiological and pathological diagnoses [14]. We conducted LSH to diagnose EPNI in the validation set and provided a pathological label. The results indicated that the diagnostic accuracy based on radiological signs was 87.5%. Subgroup analysis showed that the accuracy was 66.6% when EPNI presented as a streaky and strand-like intensity structure, and 92.3% when it presented as irregular soft tissue, which was similar to previously reported results. In diagnosing EPNI, our model performed well, with an accuracy of 79.2% in the training set and 72% in the validation set. Further analysis demonstrated that the false-positive rate was the major factor affecting the efficacy of our model, with regional adipose tissue inflammation and obstructive pancreatitis potentially contributing to this outcome. Regarding the prediction accuracy for different targeted areas, the model also demonstrated a favorable discriminative ability.

In this study, we introduced an attention mechanism to enhance the performance of nnUNet [35]. This prompted the model to focus on segmenting the neural plexus between the pancreas and arteries, which contributed to the subsequent precise prediction of EPNI. However, our study has some limitations. Firstly, since these cases were collected from a single center and were retrospective in nature, bias was inevitable. To confirm the model’s generalizability in future multicenter studies, we plan to expand the validation cohort with more diverse datasets to address potential distribution shifts and incorporate external validation sets from independent institutions. Secondly, as previously mentioned, the ground truth labels in the training set were based on radiological diagnoses. Therefore, the model can only be considered preliminary, even though it has been tested with pathological labels in the validation set. Large sample sizes with pathological labels and multicenter studies are required to improve the efficacy of our model. Thirdly, the aim of our study was to develop a fully automatic model, and relevant clinical data were not collected, which may have significant implications for predicting EPNI. Hence, related work is required to improve the prediction performance of the model in the future. Lastly, the distribution of cases in the training and validation set may pose a risk of overfitting in the results. To mitigate overfitting in future multicenter studies, we will employ several strategies during model development, such as data augmentation to generate more training samples and help the model learn more generalized features, regularization techniques such as L2 regularization and dropout layers in the neural networks, and cross-validation during hyperparameter tuning to ensure robustness.

In conclusion, our deep learning model demonstrated favorable performance in segmenting the extrapancreatic nerve plexus and diagnosing EPNI. Nevertheless, further prospective and multicenter studies are required to improve and assess the performance of this model.

## Supplementary Material

Supplementary material.docx

## Data Availability

The data that support the findings of this study are available from the corresponding author, [Liang Mao, E-mail: maoliang@njglyy.com], upon reasonable request.
