# Supplementary material for: Deep learning to predict extrapancreatic perineural invasion at CT images
Source: Ann Med. 2025 Dec 12;57(1):2568116. doi: 10.1080/07853890.2025.2568116 (PMC12704129; doi:10.1080/07853890.2025.2568116)
Supplement: Supplementary material.docx [file IANN_A_2568116_SM9554.docx]

**Supplementary Files**

1. **Supplementary Method**

**Large section of histopathology (LSH) and pathological image analysis**

Because the assigned labels in training set were evaluated in CT images, while the golden standard for EPNI diagnosis was pathological results. However, the tissue deformation when fixed in formalin and localized EPNI make it hard for routine pathological examination to diagnose EPNI accurately. Thanks to the LSH technique, we can observe the invasive status of nerve plexus successively. The procedures of LSH are listed as follows: (a) Staining: each surgical resection margin of PD specimen are stained with ink to better differentiate them after sliced. (b) Fixation: The specimen is soaked in the formalin solution about 24 to 36 hours. (c) Slicing: Cut the pancreas into tissue blocks with a thickness of 5 millimeters perpendicular to the descending part of the duodenum. (d) Making slides: After a series procedures including dehydration, paraffin embedding, histologic slicing and HE staining, the LSH slides are made. (e) Diagnosis: EPNI around CA and SMA is diagnosed when the nerve fibers are infiltrated or surrounded more than 1/3 circumference by PDAC cells. Diagnosis of EPNI around CHA relies on the histological sections of its corresponding lymph nodes.

1. **Supplementary Figures**


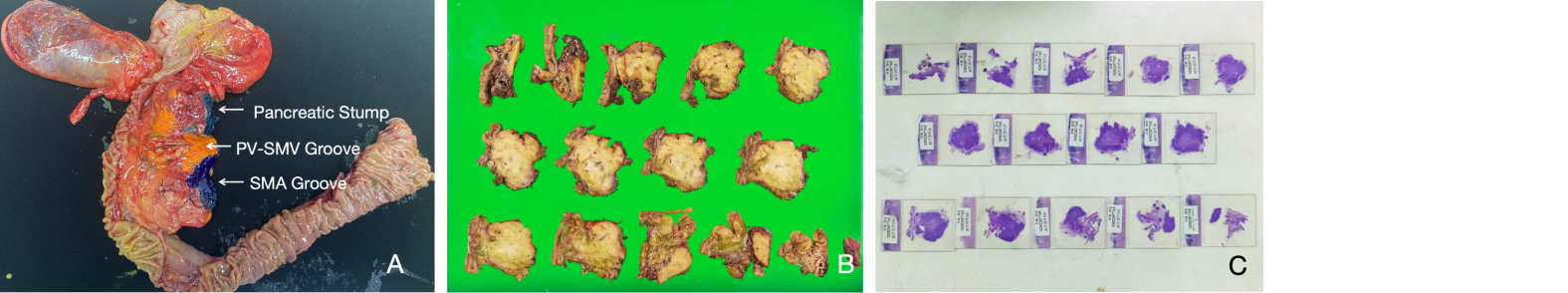


**Figure 1**: Procedures of large section of histopathology. Figure A, B, C represent specimen staining, slicing and making slides, respectively.


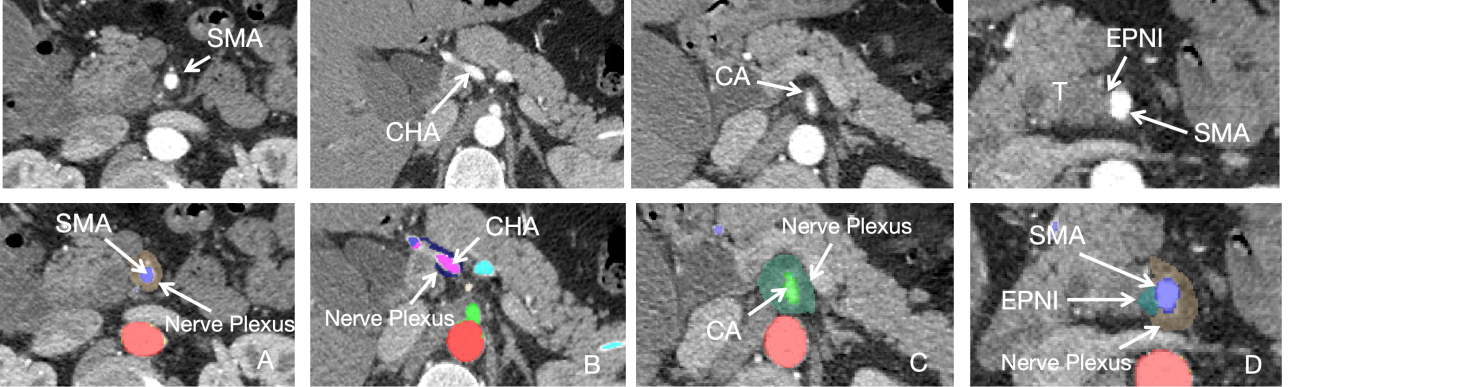


**Figure 2**: Representative CT images of manual segmentation. A, B and C illustrated the annotation of extrapancreatic nerve plexus around SMA, CHA and CA; D showed the EPNI area.


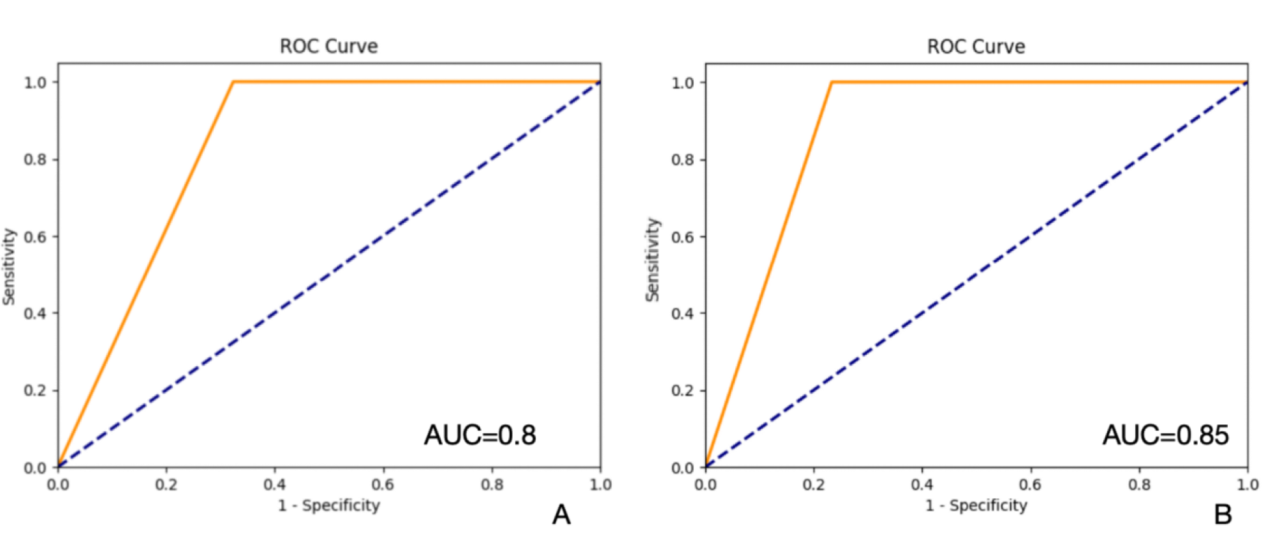


**Figure 3:** Receiver operating characteristics (ROC) curve showed the performance of deep learning model in diagnosing EPNI in training set (A) and validation set (B).
